# Comparative social costs of six early years disadvantages: a birth cohort microsimulation study

**DOI:** 10.1136/jech-2025-225472

**Published:** 2026-07-02

**Authors:** Shrathinth Venkatesh, Ieva Skarda, Aase Villadsen, Matthew Warburton, Liina Mansukoski, Mark Mon-Williams, Richard Cookson

**Affiliations:** 1 Centre for Health Economics, University of York, York, UK; 2 Centre For Longitudinal Studies, UCL Social Research Institute, London, UK; 3 School of Psychology, University of Leeds, Leeds, UK; 4 Hull York Medical School, University of York, York, UK; 5 Bradford Institue of Health Research, Bradford Teaching Hospitals NHS Foundation Trust, Bradford, UK; 6 Saw Swee Hock School of Public Health, National University of Singapore, Singapore

**Keywords:** LONGITUDINAL STUDIES, PUBLIC HEALTH, EDUCATION, QUALITY OF LIFE, CHILD HEALTH

## Abstract

**Background:**

Policymakers lack comparative information about the social costs of specific early disadvantages. We compared six policy-relevant childhood (0–5 years) disadvantages and their diverse social costs over 17 years for the UK and Bradford (a multi-ethnic district of about 550 000 in the northeast of England with high levels of deprivation).

**Methods:**

Using a microsimulation model, we compared the harms to well-being (proxied by parent-reported emotional problems), health and educational attainment and government spending (such as inpatient NHS costs) up to age 17 of the following: having a teenage mother, preterm birth, low birthweight-for-gestational-age, low height-for-age-and-sex (age 5), disability (age 5) and cognitive delay (age 5). We modelled a UK birth cohort born in 2000 and a Bradford cohort born in 2019 based on local prevalence data, uprated to 2023 prices and discounted at 3.5%.

**Results:**

Disability imposed the highest per-child cost, with a well-being loss of 7.2 WELLBYs (CI 6.1 to 8.2; monetised value £89 k) and public cost £64 k (CI £37 k–£91 k) per child by age 17. Cognitive delay had a smaller per-child loss of 3.8 WELLBYs (3.2–4.5), but the largest population-level loss was in both the UK and Bradford at 728K WELLBYs (600 K–856K; monetised value £9,044 m) and £5,588 m (£1,872-£9,303) public cost per cohort of 679 000 children born in the UK. Monetised well-being loss is almost always higher than public cost and sometimes more than twice as high.

**Conclusions:**

Cognitive delay at 5 years of age imposed larger total social costs than the other early disadvantages. For an individual child, however, well-being loss was most affected by disability or having a teenage mother.

**WHAT IS ALREADY KNOWN ON THIS TOPIC**

- Early childhood disadvantages can have substantial long-term costs to society, but few studies have captured both human costs and fiscal costs for public budgets.

**WHAT THIS STUDY ADDS**

- We compare human and fiscal costs up to age 17 across six specific early childhood disadvantages, including five core adolescent educational and health outcomes with lifelong consequences for well-being and public cost.
- Cognitive delay was the costliest of the six early childhood disadvantages in terms of population total well-being loss, public cost and harm to educational attainment, but disability and having a teenage mother were more harmful at an individual child well-being level.

**HOW THIS STUDY MIGHT AFFECT RESEARCH, PRACTICE OR POLICY**

- The findings support policies that target improved school readiness as a way of breaking the links between a child’s background and life chances at a population level.

## Introduction

 Early life disadvantages, from conception to 5 years of age, have numerous long-term social costs, including financial costs for public budgets as well as human costs to people’s health, well-being and education.^1^ However, little is known about the relative magnitude of these costs for specific early life disadvantages*,* and previous studies have not used standardised methods to compare the social cost of different disadvantages by monetising well-being and calculating cross-sectoral public costs. This information is crucial for policymakers seeking to set priorities for efficient investment in prevention and support for early childhood disadvantages. A simulation model that combines different sources of data (eg, LifeSim Childhood^2^) can provide such evidence for policymakers.

We compared the long-term social costs of six early disadvantages that are often the target of local and central government prevention and family support policy interventions and are known to have important long-term human and financial costs. These are as follows: having a teenage mother, preterm birth, low birth weight, low height at age 5 years, disability at age 5 years and cognitive delay at age 5 years. Each of these risk factors has been shown to be associated with poor outcomes in childhood and adulthood.^3–7^ We present the effects on adverse outcomes and the cumulative costs to the UK government at age 17 years.

This study makes four main contributions. First, it provides the first systematic comparison of well-being, health, educational and public costs across multiple early life disadvantages. Second, it integrates well-being valuation using well-being-adjusted life years (WELLBY) with public sector cost estimates, allowing a unified assessment of social costs. Third, it demonstrates the application of national modelling to a local, multi-ethnic and deprived context, offering a transferable framework for evidence-informed early years policy for both national and local government. Fourth, it illustrates the importance of connecting administrative datasets and evaluating interventions across multiple public services at both a national and a ‘place-based’ level.

Our main results focus on a UK birth cohort of 679 029 children born around 2000. We also present a simple illustrative example of how our findings can be translated to more recent birth cohorts in specific localities (in this case, a cohort of 7200 children born in Bradford in 2019) and highlight the implications for policies around the UK’s Opportunity Mission, a Labour government policy announced in 2024 to give children the best start in life and break the link between a child’s background and their future success, including a milestone for 75% of 5 year olds to reach a good level of development in language, maths and literacy and personal, social and emotional development by 2028.

## Methods

### Study design and population

This was a microsimulation modelling study that used a published model (LifeSim Childhood^2^) to estimate long-term social costs for a national UK cohort of children, with probabilistic sensitivity analysis to handle uncertainty (online supplemental appendix figure A1 provides an overview of the structure of the LifeSim Childhood model). The UK cohort was based on the Millennium Cohort Study (MCS) of children born from 2000 to 2001. We also used local administrative data containing population demographics and prevalence to translate those findings to a cohort of all children born in the Bradford District in 2019.

### Data

Both the national population sample and the causal effect parameters in our simulation were estimated from the MCS, a nationally representative prospective cohort study following 19 517 children born in the UK between September 2000 and January 2002.^8^ The surveys were administered when the children were 9 months, 3, 5, 7, 11, 14 and 17 years. Ethics approval for the MCS study was obtained through the National Health Service Research Ethics Committee system with written informed consent up to age 14 years given by parents and verbal consent from those aged 16 years and older. Additional details on MCS can be found elsewhere.^9^

We addressed missing data in the MCS (primarily due to attrition) using multiple imputation with chained equations.^10^ Imputation was carried out from the 3-year age survey (sweep 2). Our primary imputation sweep, which included 692 additional families not surveyed in sweep 1. Our primary analysis dataset included observations on 15 380 children after imputation of 50 datasets. Further details of the imputation are in online supplemental appendix note A1, and descriptive statistics on the pooled imputed datasets are in online supplemental appendix table A1.

We also looked at the effects for Bradford district in the north of England, which is the 12th most deprived local authority in England and where more than 40% of children live in poverty. Bradford district is also ethnically diverse, with nearly two-fifths of the population identifying as non-white, including a substantial Pakistani community making up just over a quarter of the population. For this, we used data from Connected Bradford, a research database comprising administrative data for the Bradford district.^11^ Ethical approval for this data was provided by the Leeds Bradford Research Ethics Committee for Connected Bradford IRAS ref: 239924, CAG ref: 18/CAG/0091 and REC ref: 18/YH/0200. Specifically, we used the latest available pre-pandemic data from three administrative datasets: birth records from the Bradford Royal Infirmary hospital in 2018 and 2019 (for the prevalence of the three birth risk factors) and the early years foundation stage profile (EYFSP) and National Child Measurement Programme (NCMP) during the 2017/2018 and 2018/2019 academic years (around age 5) for cognitive delay and low height, respectively. The Bradford prevalence rates are thus based on a year 2018–19 birth cohort for having a teenage mother, preterm birth and low birth weight and a 2012–2014 birth cohort (age 5 in 2017–19) for low height and cognitive delay. The hospital birth data contain circa 5000 births and the 5-year-old school records contain circa 10 000 children; we could not obtain the precise numbers due to the risk of confidential data being inadvertently disclosed to the research team through a process of reverse engineering.

For calculating the full effect for the UK we assumed a birth cohort of 679 029 (The number of live births in the UK in 2000 based on Vital statistics dataset) from the Office for National Statistics. Our calculations for Bradford assumed a birth cohort of 7200 based on the number of births in the district in 2019 as reported in the City of Bradford Metropolitan District Council Intelligence Bulletin on population projections from March 2020.^12^

### Measures

#### Early years disadvantages

Our analysis focused on the effect of six ‘early years’ disadvantages from birth to age 5 years using the MCS.

The first risk factor was having a teenage mother, based on the mother’s reported age at the time of birth, dichotomised (<=19 years). The second risk factor was preterm birth, based on the number of weeks of gestation at the time of birth reported by the parent, dichotomised (<=37 weeks). The third risk factor was low birth weight for gestational age, based on parent reported weight of the child in grams compared with percentiles in the UK birth weight chart,^13^ dichotomised (being in the bottom 10th percentile). The fourth risk factor was low height for age, based on the height of the child measured at age five compared with percentiles in the UK height chart for age in months and sex,^13^ dichotomised (being in the bottom 10th percentile). The fifth risk factor was disability, based on parent reporting of the child having a long-term illness which limits their day-to-day activity. The final risk factor was cognitive delay or ‘learning difficulties’ at age 5 years, based on the child’s performance in three British Ability Scales (BAS) II test, naming vocabulary, pattern construction and picture similarities,^14^ dichotomised (being in the bottom 30th percentile).

#### Health and educational outcomes at age 17

We examined five adverse health and educational outcomes at age 17 years that are known to have important life-long impacts on health, well-being and income in adulthood.^15^ The first measure was psychological distress, which was self-reported by the participants using the six-item Kessler scale.^16^ We used the clinically determined threshold (≥13) as indicating the presence of psychological distress.^16^ The second measure was self-rated health, which was assessed by asking ‘How would you describe your health generally?’. We classified responses of excellent, very good and good as being in good health, and responses of fair and poor as being in poor health. The third measure was obesity. This factor was based on the adolescents’ weight and height, taken by the interviewer at the participant’s home, and classified using the obesity threshold from the British 1990 (UK90) growth reference chart for children.^13^ The fourth measure was regular cigarette smoking (excluding e-cigarettes), which was self-reported smoking of more than six cigarettes per week. The fifth measure was poor academic achievement based on self-reported exam results at the end of secondary school, that is, General Certificate of Secondary Education (GCSE) results in England, Wales and Northern Ireland, and National 4 and National 5 (N5) results in Scotland. Poor academic attainment was classified as not achieving five or more GCSEs, including in maths and English, graded C or above, or five or more N5s, including in maths and English, graded D or above.

#### Well-being from age 3 to 17

Parents of the MCS children reported the Strengths and Difficulties Questionnaire (SDQ) emotional problems scale for the child between 3 and 17 years of age. This score was mapped linearly to a life satisfaction scale between 2 and 10. We translated a change in life satisfaction on this scale to a WELLBY, a single unit change in life satisfaction (on a 0 to 10 scale) per person per year, which is valued by the treasury at £15,450 in 2023 (Estimates of the well-being effects based on life satisfaction measured using SDQ Internalising are presented in online supplemental appendix table A6).^17^

#### Cost-bearing outcomes

Parents reported six outcomes for the children between 3 and 17 years of age that are associated with public costs (online supplemental appendix table A2 summarises the costs and cost bearing outcomes used).

The first, hospitalisation, was based on a parent report of the child being admitted to a hospital in the past year between ages 3 and 17 years. This was calculated as a single inpatient visit for a child in the UK (£1,587).^18^ The second, disability, is based on parent-reported long-term illness that affects the child’s daily activity in the past year between ages 3 and 17 years (disability is still a cost bearing outcome when disability at age 5 is the risk factor as we do not estimate the effect of disability on future disability but rather on future disability costs). This cost is the average annual cost of a child to the NHS (£1,009).^19^ The third, 'Conduct Disorder’, based on the scores derived from the parent reported version of the Strengths and Difficulties Questionnaire (SDQ) conduct problems scale between ages 3 and 17 years. This is based on annual cost to the department of education, NHS and social services at different ages (£3,092 between ages 5 and 10, £1,963 between ages 11 and 16 and £235 at age 17).^20^ The fourth, special educational needs (SEN), was based on parent report of the child having a statement of special educational needs between ages 7 and 14 years, costed at the average cost of EHC Plan in England in 2023 (£25,500).^21–23^ The fifth, truancy, based on parent reports of any unexcused absence from school in the last year at ages 11 and 14 years. This is costed based on the proportion of regular truancy among those with any truancy and annual cost of regular to the department of education from truancy (£943).^24^ Finally, exclusion, based on parent reports of any exclusion in the past year from school ages 11 and 14 years. This is costed based on the proportion of those permanently excluded among those with any exclusion and the cost of alternative provision for a permanently excluded student (£21,848).^25^

### Causal inference

We used a microsimulation model that relies on a causal inference approach based on causal diagrams,^26^ as described previously.^2^ In brief, LifeSim Childhood estimates causal relationships between each early years risk factor and outcome using observational longitudinal data, controlling for a rich set of observable confounders. We use a separate model for each risk factor where we controlled for confounders where there is a plausible justification based on current scientific knowledge and a set of causal inference rules that are described and justified elsewhere.^2^ The set of confounders varied appropriately by risk factor, as described in online supplemental appendix note A2 and a simplified DAG is presented in online supplemental appendix figure A2. For all risk factors, we controlled for country and region of birth, ethnicity and Index of Multiple Deprivation (IMD) quintiles. We also selected further confounders, as appropriate, from the following list: sex, age of parent, permanent household income, maternal smoking during pregnancy, parental disability, grandparents’ education.

We used negative binomial regressions for count outcomes (SDQ scores and Kessler) and logistic regression for binary outcomes. Analyses were weighted to adjust for sampling design and for attrition between the birth sweep and the age 3 years sweep, which was the multiple imputation sample.

### Simulation modelling

We simulated the effect of each of these risk factors separately using LifeSim Childhood. A cohort of 20 000 children randomly chosen from the multiply imputed MCS data, based on UK population weights, were run through the model 100 times. The parameters for each run were based on the models estimated through regressions using the MCS. The coefficients and standard errors were used to generate a distribution of potential coefficients from which one was chosen for each run. Random error was also introduced to the final estimate by adding a stochastic error value from the distribution of errors for each model estimated (online supplemental appendix table A7 compares simulated and actual estimates for important outcomes at age 17).

## Results

Table 1 shows the estimated prevalence of each of the six ‘early years’ disadvantages in the UK and Bradford (where prevalence rates are generally slightly higher than the UK). Cognitive delay had the highest UK prevalence of 27.7%, based on a 30th percentile cut-off to match school readiness figures, with preterm birth, low birth weight and low height all around 10%, based on 10th percentile cut-offs, and disability and having a teenage mother at about 7.5% and 5%, respectively.

**Table 1:** Population prevalence of six early life disadvantages

Risk factor	UK MCS prevalence (Born 2000; age 5 in 2005)	Bradford prevalence (Born 2017; age 5 2017–19)
Having a teenage mother	4.95	3.37
Preterm birth	6.37	6.82
Low birth weight	13.09	12.85
Low height age 5	10.06	8.72
Disability age 5	7.44	*N/A*
Cognitive delay age 5	27.72	30.55

UK MCS data from longitudinal survey; Bradford data from administrative records. There were approximately 679 029 births in the UK in 2019 and 7200 in Bradford. It was not possible to estimate the prevalence of disability at age five within Bradford, as this is not measured in administrative data and the Born in Bradford study only asks questions about disability at a later age.

MCS, Millennium Cohort Study.

Figure 1 shows the effects of the six risk factors on poor GCSE performance and psychological distress at age 17, and the effects on all five outcomes are reported in online supplemental appendix table A3. All six risk factors have effects on GCSE performance, and disability has a significant effect on psychological distress, but the risk factors have little or no impact on the other three outcomes. Individual-level analysis (Panel A) showed the largest effects on GCSE performance were cognitive delay (21.7%) followed by having a teenage mother (15.9%). Population-level analysis (Panel B) showed cognitive delay (‘learning difficulties’) had by far the largest effect on GCSE performance due to its high prevalence. Disability slightly increased the risk of obesity and poor self-rated health, having a teenage mother slightly increased the risk of regular smoking, and low birth weight and low height at age five slightly decreased the risk of obesity at age 17 years. These were the only significant effects.

**Figure 1:**
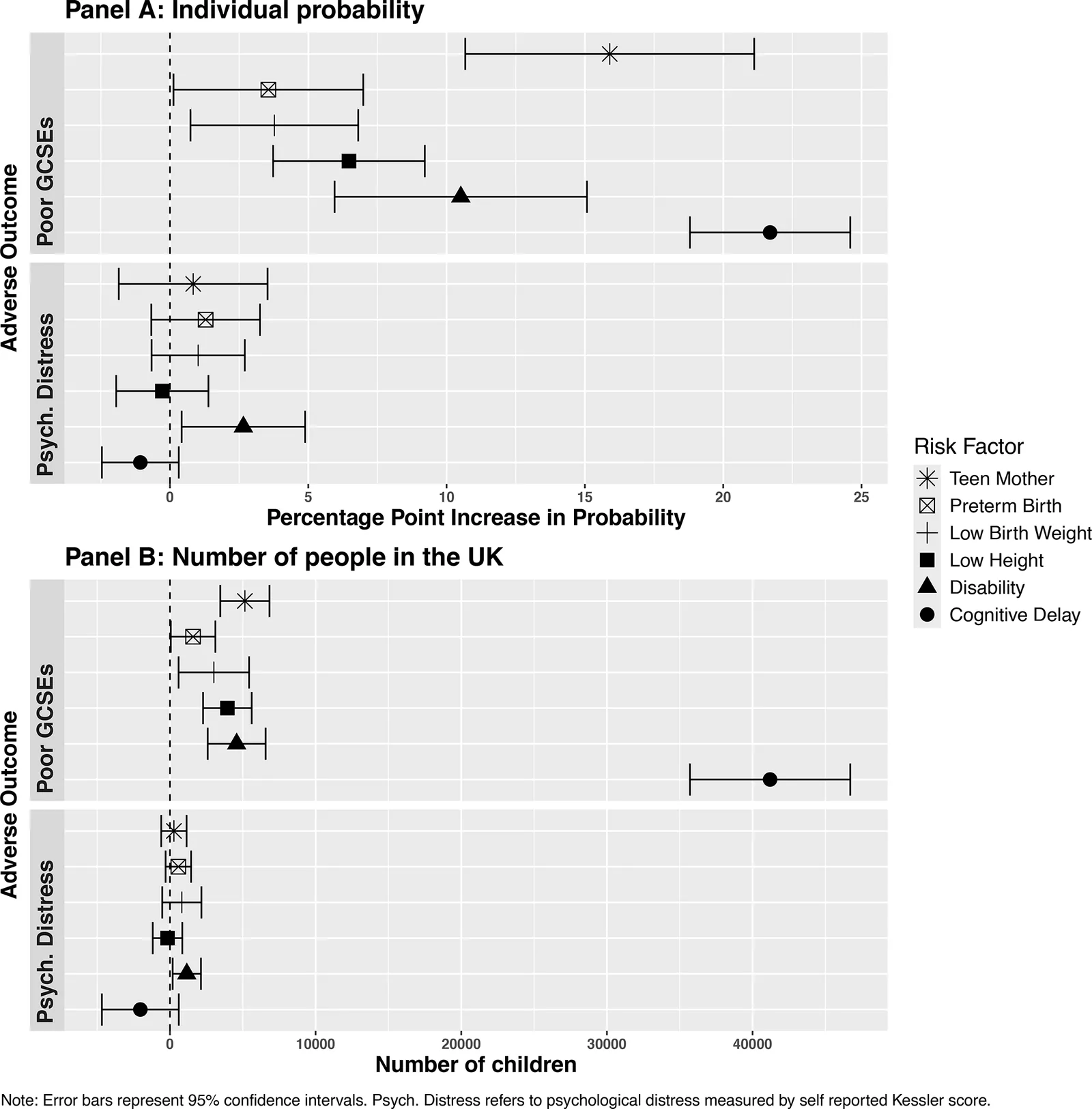
Increase in adverse outcomes at age 17 for UK 2000 birth cohort.

Figure 2 shows the effects on cumulative well-being up to age 17 years, with underpinning data in online supplemental appendix table A4). Individual-level analysis (Panel A) showed all risk factors except low birth weight had significant effects on well-being. Disability imposed a cost of 7.2 WELLBYs (6.1–8.2), 4.2 (3.3–5.2) for having a teenage mother, 3.8 (3.2–4.5) for cognitive delay, 3.1 (2.4–3.8) for preterm birth, 1.8 (1.1–2.5) for low height. UK population-level analysis (Panel B) showed cognitive delay imposed a cost of 727,996 WELLBYs (599,819-856,172), 312 933 (267,012-358,854) for disability, 1 38 937 (1 07 332–1 70 541) for pre-term birth, 137 322 (105,059-169,584) for having a teenage mother, 107 372 (65,103-49,641) for low height.

**Figure 2:**
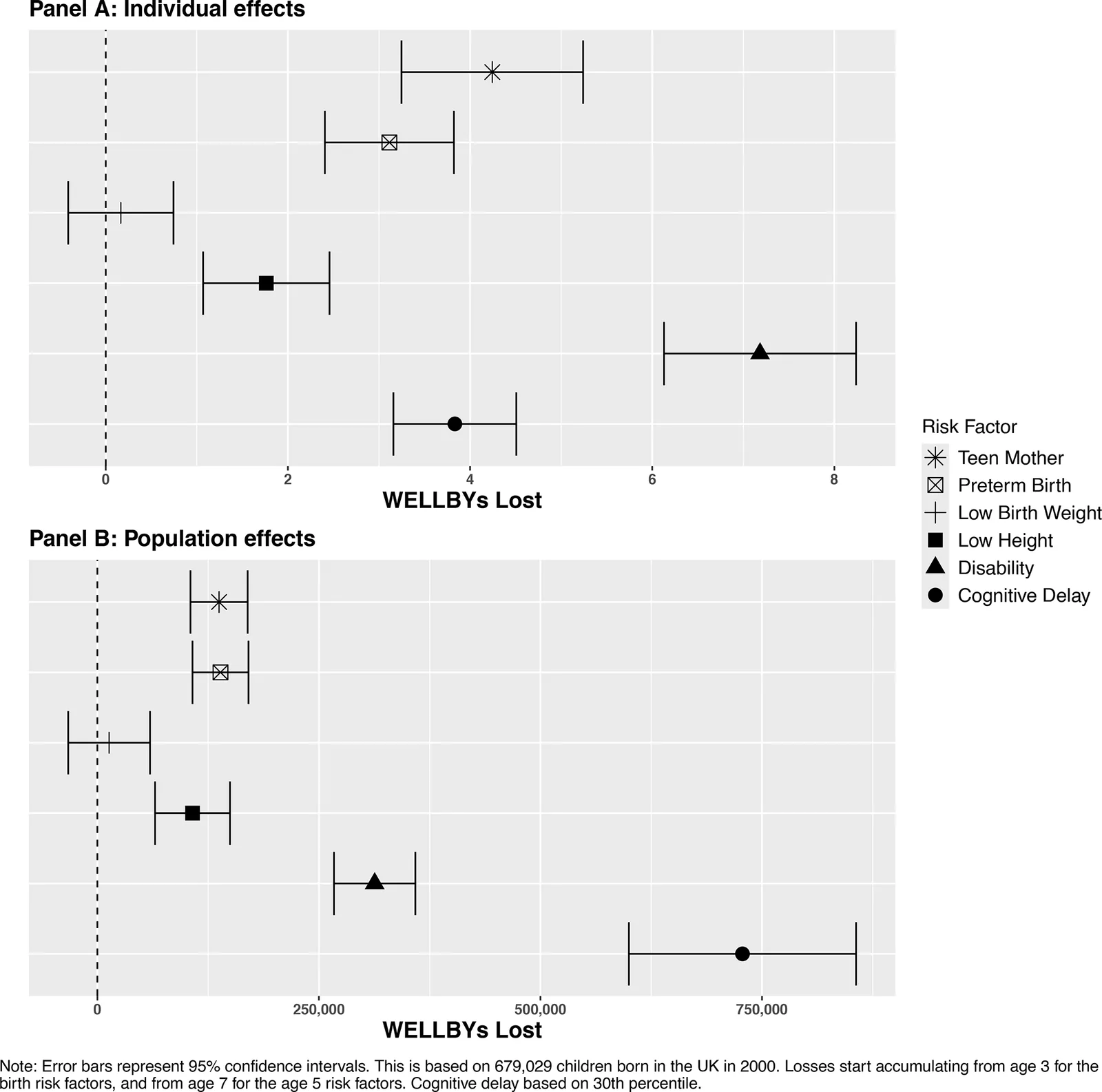
Cumulative wellbeing loss up to age 17 for UK 2000 birth cohort.

Figure 3 shows the effects on cumulative public costs up to age 17 years, with underpinning data in online supplemental appendix table A5. Individual-level analysis (Panel A) showed disability imposed a cost of £64,053 (37,423–90,683), cognitive delay £29,421 (9,859–49,984), preterm birth £12,696 (6,245–19,167), low height £11,258 (4,031–18,485), and having a teenage mother £3,0781 (104–6,058). UK population-level analysis (Panel B) showed cognitive delay imposed a cost of £5,588 m (1,872–9,303), disability £2,790 m (1,630-3,950), low height £686 m (246–1,126), pre-term birth £556 m (278-855), having a teenage mother £100 m (3-196), but low birth weight had no significant effect on public cost.

**Figure 3:**
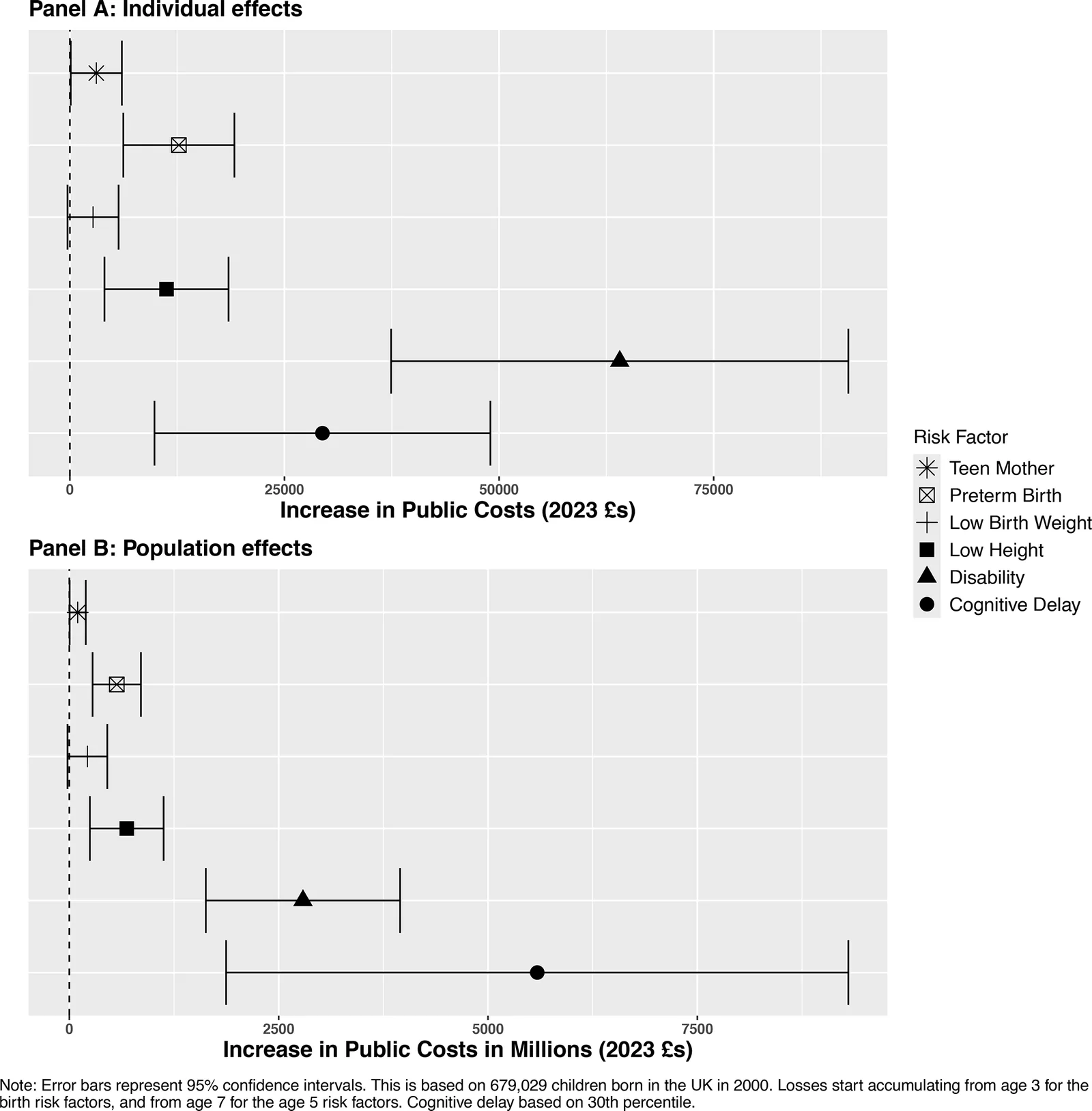
Cumulative public costs increases up to age 17 for UK 2000 birth cohort.

Table 2 presents population-level social costs for the UK and Bradford, broken down by public cost and monetary value of well-being loss, all discounted at 3.5%. For the Bradford effects, we assumed the same average individual-level effects and costs as for the UK but multiplied up to the Bradford population based on prevalence and population size. Our strategy is currently very simple and does not take into account the ethnic and socio-economic differences for Bradford. The risk factor with the highest social cost was cognitive delay, valued at £14,632 m in the UK and £169 m in Bradford. The next highest social costs were imposed by disability (£6,654 in the UK) and pre-term birth (£2,081 m in the UK, £23 m in Bradford). The monetary value of well-being loss was almost always higher than the public cost, usually more than 50% higher, and sometimes more than twice as high (the six ratios derived from table 2 UK figures were 0.61, 1.38, 1.62, 1.90, 2.67 and 15.94).

**Table 2:** Population total public cost and well-being loss in the UK and Bradford

		Having a Teen Mother	Preterm birth	Low birth weight	Low height	Disability	Cognitive delay
Wellbeing and costs total for UK 2000 birth cohort							
*Costs*	Total Costs (2023, £m)	99.70	566.40	215.82	685.71	2790.15	5587.84
well-being	WELLBYs	137 322	138 937	13 242	107 372	312 933	727 996
	Monetary value (2023, £m)	1590	1515	133	1304	3864	9044
*Total*	Monetary value (2023, £m)	1689	2081	349	1990	6654	14 632
Wellbeing and costs total for Bradford 2019 birth cohort							
*Costs*	Total Costs (2023, £m)	0.75	6.23	2.51	7.07		64.72
Well-being	WELLBYs	1030	1529	153	1107		8431
	Monetary value (2023, £m)	11.92	16.68	1.54	13.44		104.74
*Total*	Monetary value (2023, £m)	12.67	22.91	4.04	20.51		169.46

UK MCS data from longitudinal survey; Bradford data from administrative records. There were approximately 679 029 births in the UK in 2019 and 7200 in Bradford. It was not possible to estimate the prevalence of disability at age five within Bradford, as this is not measured in administrative data and the Born in Bradford study only asks questions about disability at a later age. A WELLBY is a unit increase in a 0 to 10 life satisfaction scale over a year and if valued by HM Treasury at about £15,450 in 2023. All monetary values are discounted at 3.5%.

MCS, millennium cohort study; WELLBY, well-being-adjusted life years.

## Discussion

### Main findings of this study

Cognitive delay (‘learning difficulties’) produced the largest population total social cost from the six risk factors studied, though disability imposed a larger well-being loss to the individual child. Cognitive delays were also associated with the highest likelihood of poor performance in GCSEs, followed by disability and having a teenage mother. Disability imposed the largest public cost per case, but cognitive delay is more prevalent and thus had the largest total cost at a population level. All the risk factors except low birth weight imposed significant well-being costs on the individual child, but most had little or no impact on psychological distress, obesity, self-reported health or smoking. The monetary value of well-being loss was almost always higher than the public cost and sometimes more than twice as high.

### Strengths

A major strength of our study is the ability to compare the relative magnitude of the social costs of different early life disadvantages, using a common dataset and methodological approach. This is important because most costing studies focus on one risk factor at a time, hindering comparison and making it hard for policymakers to assess priorities. In addition, the use of high-quality longitudinal data following the lives of more than 15 000 Gen Z individuals from birth to age 17 years allowed causal inference based on a risk set of confounding variables. A third strength is our use of simulation modelling to synthesise evidence from different sources and to address different sources of uncertainty simultaneously using probabilistic sensitivity analysis. A final strength is our use of connected place-based administrative data from a District (Bradford) to illustrate how national modelling can be used to inform local decision-making that takes the costs across the system into account when determining the most cost effective strategies to improving outcomes for children and young people.

### Limitations

One limitation is that our estimates of impacts on child and adolescent well-being rely on the proxy measure of parent-reported SDQ emotional problems score. This was the only consistent way of measuring well-being from age 3 years using MCS data, as self-report measures are only available from age 11 years and vary across different survey waves.^2^ This could mean that we slightly overestimate the well-being losses in absolute terms since parent-reported well-being levels tend to be slightly higher than self-reported levels during adolescence. Another limitation is that our public cost estimates are conservative underestimates. We miss public costs for rare but costly events (eg, being taken into care and youth justice costs), which are hard to measure using survey data due to sample size and data sensitivity issues, and our cost estimates for social care costs associated with disability and exclusion are incomplete. We also do not include spillover costs for parents, siblings, classmates, businesses and communities. Another limitation is that despite controlling for a rich set of observable confounders, our causal estimates may nevertheless be biased due to confounding by unobserved factors (eg, genetically heritable traits and parental personality traits). Another limitation is that we estimate causal effects for Gen Z, born 2000/01, but then apply these to Gen A, born today. There is therefore a risk of cohort bias in our estimates, due to generational change. Another limitation is that we are unable to properly model the covariance of all our outcomes due to the large number and variety of estimation strategies used for each outcome. This may lead to underestimation of overall uncertainty.

It is also important to note that the costs of each risk factor are not straightforwardly additive due to the risk of double counting. In most cases we control for the other risk factors, but not all of them and there is also risk of double counting due to interactions between the mediating pathways. We therefore do not estimate the benefit from eliminating multiple disadvantages simultaneously, as this would likely be an overestimate. A final limitation is that we do not extrapolate the costs further into adulthood. Poor educational outcomes are associated with worse outcomes throughout adulthood suggesting that the lifetime costs of cognitive delay and other risk factors are far larger than those captured in this paper.

### Research and policy implications

Our results focus on microeconomic estimation of the causal effects of interventions on national expenditure and outcomes rather than macroeconomic forecasting of future levels of national expenditure and outcomes. We therefore do not attempt to forecast the effect of macroeconomic shocks like COVID on population mean outcomes. However, we recognise that shocks to the macroeconomic environment may potentially modify the causal effects of interventions, and this is a limitation of our modelling. Policies aimed at decreasing the number of children arriving at school with cognitive delay appear to be highly justified relative to policies addressing other forms of disadvantage. A key factor might be better and more affordable childcare provision for disadvantaged families, with research showing that high-quality universal childcare attendance significantly improves school readiness, and this positive effect is amplified for children from the most disadvantaged backgrounds.^27 28^ Thus, our findings provide evidence in support of the government’s recent expansion of free childcare and its Opportunity Mission. Our findings also broadly support the importance of the UK government’s current aspiration for 75% of children to achieve a good level of development at primary school entry, a key component of which involves children having the necessary cognitive skills for learning.^28^ However, disability and having a teenage mother have larger impacts on child well-being, and decision makers should not lose sight of these additional issues when trying to break the links between a child’s background and life changes (the stated ambition of the UK’s Opportunity Mission).

## Supplementary material

10.1136/jech-2025-225472 online supplemental file 1

## Data Availability

Data are available in a public, open access repository.
