# Supplementary material for: Comparative social costs of six early years disadvantages: a birth cohort microsimulation study
Source: J Epidemiol Community Health. 2026 Jul 2;80(10):e225472. doi: 10.1136/jech-2025-225472 (PMC13638340; doi:10.1136/jech-2025-225472)
Supplement: Supplementary file 1 [file jech-80-10-s001.docx]

**Supplementary material for** **“Comparative social costs of six early years disadvantages: A Birth cohort microsimulation study”**

**Appendix Note A1: Multiple imputation approach**

Of the cohort of 19,519 children in the MCS only 10,757 children provided any data at age 17, this is without accounting for incomplete responses to specific questions even in years they did participate. To deal with this attrition and missingness in the data we use multiple imputation. We believe this helps increase the statistical power and reduce bias in our estimation of effects.

We used chained equations to impute missing data back to the second sweep (age 3) of the MCS. We use the second sweep instead of the first as more than a thousand eligible households were added to the cohort in that sweep that were missed in the first sweep. We follow Villadsen et al. (2023) and include a number of the same auxiliary variables that are predictive of the outcomes at age 17 and earlier in the imputation to improve the accuracy of our estimates. These variables are from various sweeps and included child physical health, parental substance use, maternal mental health, and other household characteristics. We generate 50 datasets using multiple imputation. Post-imputation, the final sample used in our analyses consists of 15,380 cohort members.

**Appendix Note A2: Confounding variables**

To estimate the relationship between each of the six risk factors and all the outcomes we identify confounders, i.e. factors that influence both the risk factor and the outcome. By controlling for these confounders, we hope to estimate the causal effect of the risk factor on the outcomes. We believe the detailed information in the MCS data allows us to capture nearly all confounders in the relationship between the outcomes and risk factor. However, we do not have access to genetic data, and genetics may be a confounding factor for some of these risk factor-outcome combinations. We attempt to correct for this by including some variables that may capture some genetic traits.

Below we list the confounders we use in our models for each risk factor. For this paper we do not vary the confounder list by outcome for the sake of simplicity and we do not believe that controlling for variables that are neither mediators nor moderators will affect the estimation of the model.

1. Teen Mother

For estimating the effect of having a teenage mother on outcomes we control for country of birth (indicators for Scotland, Wales and Northern Ireland, England as baseline), region in England (indicators for North East, North West, Yorkshire and the Humber, East Midlands, West Midlands, East of England, London, South East, and South West, with London as baseline), ethnicity (indicators for mixed, Indian, Pakistani and Bangladeshi, Black or Black British, and other ethnic group (inc. Chinese and other Asian, with white as baseline), IMD quintile (indicators for bottom four IMD quintiles, highest IMD quintile as baseline) and grandparent’s education (indicators for Grandparent’s NVQ education level, highest education level as baseline).

2. Pre-term birth

For estimating the effect of pre-term birth on outcomes we control for country of birth (indicators for Scotland, Wales and Northern Ireland, England as baseline), region in England (indicators for North East, North West, Yorkshire and the Humber, East Midlands, West Midlands, East of England, London, South East, and South West, with London as baseline), ethnicity (indicators for mixed, Indian, Pakistani and Bangladeshi, Black or Black British, and other ethnic group (inc. Chinese and other Asian, with white as baseline), IMD quintile (indicators for bottom four IMD quintiles, highest IMD quintile as baseline), child sex (indicator for male, female as baseline), mother smoking during pregnancy, log household income (log OECD equivalised weekly household income) and mother’s age at birth (indicators for teenage mother and for mother over 35).

3. Low Birth Weight

For estimating the effect of low birth weight on outcomes we control for country of birth (indicators for Scotland, Wales and Northern Ireland, England as baseline), region in England (indicators for North East, North West, Yorkshire and the Humber, East Midlands, West Midlands, East of England, London, South East, and South West, with London as baseline), ethnicity (indicators for mixed, Indian, Pakistani and Bangladeshi, Black or Black British, and other ethnic group (inc. Chinese and other Asian, with white as baseline), IMD quintile (indicators for bottom four IMD quintiles, highest IMD quintile as baseline), mother smoking during pregnancy, log household income (log OECD equivalised weekly household income)  and mother’s age at birth (indicators for teenage mother and for mother over 35).

4. Low Height

For estimating the effect of low height on outcomes we control for country of birth (indicators for Scotland, Wales and Northern Ireland, England as baseline), region in England (indicators for North East, North West, Yorkshire and the Humber, East Midlands, West Midlands, East of England, London, South East, and South West, with London as baseline), ethnicity (indicators for mixed, Indian, Pakistani and Bangladeshi, Black or Black British, and other ethnic group (inc. Chinese and other Asian, with white as baseline), IMD quintile (indicators for bottom four IMD quintiles, highest IMD quintile as baseline), mother smoking during pregnancy, and log household income (log OECD equivalised weekly household income).

5. Disability

For estimating the effect of disability on outcomes we control for country of birth (indicators for Scotland, Wales and Northern Ireland, England as baseline), region in England (indicators for North East, North West, Yorkshire and the Humber, East Midlands, West Midlands, East of England, London, South East, and South West, with London as baseline), ethnicity (indicators for mixed, Indian, Pakistani and Bangladeshi, Black or Black British, and other ethnic group (inc. Chinese and other Asian, with white as baseline), IMD quintile (indicators for bottom four IMD quintiles, highest IMD quintile as baseline), sex, mother’s age at birth, mother smoking during pregnancy, maternal malaise (indicator for rutter score greater than or equal to 4), disability in household (indicator for either parent in the house being disabled), and log household income (log OECD equivalised weekly household income).

6. Cognitive delay

For estimating the effect of cognitive delay on outcomes we control for country of birth (indicators for Scotland, Wales and Northern Ireland, England as baseline), region in England (indicators for North East, North West, Yorkshire and the Humber, East Midlands, West Midlands, East of England, London, South East, and South West, with London as baseline), ethnicity (indicators for mixed, Indian, Pakistani and Bangladeshi, Black or Black British, and other ethnic group (inc. Chinese and other Asian, with white as baseline), IMD quintile (indicators for bottom four IMD quintiles, highest IMD quintile as baseline), sex, mother’s age at birth (indicators for teenage mother and for mother over 35), birth weight (indicators for low, or high birth weight for gestational age), gestational age (indicators for preterm birth, very preterm birth and extreme preterm birth, baseline is gestational age  greater than 37 weeks), mother smoking during pregnancy, having a single parent, and log household income (log OECD equivalised weekly household income).

**Appendix Note A3: Cognitive delay measurement in MCS**

We used cognitive delay or “learning difficulties” at age 5 years as a proxy for the official measure of school readiness used by the UK government, based on the teacher-reported early years foundation stage profile (EYFSP). The official binary measure of a “good level of development” is more holistic than ours, as it includes not just cognitive skills but also non-cognitive skills including social, emotional, behavioural and motor skills. However, this official measure was not included in the publicly available MCS data. Cognitive delay in our study was based on the child’s performance in three British Ability Scales (BAS) II tests, Naming vocabulary, Pattern construction and Picture similarities. The age adjusted z-scores for the results of each of these tests were added and standard normalised once again. We used the bottom 30% as a cut-off for our proxy measure as the proportion of students with delayed school readiness on the official measure was about 30% for the cohort of children born around 2000 and was still only 32% in our simulation start year of 2023. (Department for education, Early years foundations stage profile results 2024/2025)

**Appendix Note A4: Wellbeing measurement and valuation**

**Wellbeing measurement**

We measure wellbeing using a simple linear range-based mapping of parent-reported Strengths and Difficulties Questionnaire (SDQ) scores. For ages 3 to 17, we utilize the SDQ Emotional Symptoms subscale (measured on a 0–10 scale). Because a higher SDQ score is associated with lower wellbeing, we convert these scores into a Life Satisfaction (LS) score. Specifically, we map the results to a range between 2 and 10 using the following formula:

${LS}_{i}=2+ \frac{{10- SDQes}_{i}}{10}\times8$

where ${LS}_{i}$ is life satisfaction and ${SDQes}_{i}$ is parent reported SDQ emotional symptoms score.

We start our life satisfaction range at 2 rather than 0 for two reasons. First, when calculating WELLBYs, a score of 0 or 1 is interpreted by HM Treasury as “worse than death" and life satisfaction studies find that only a very small portion of children ever actually record scores in that range - smaller than the proportions in our MSC study with SDQ ES scores of 10 or 9. Second, restricting the range in this way is conservative: it reduces our estimated causal effects. A linear mapping across the full 0–10 spectrum would thus not only result in a substantial and inaccurate proportion of children falling below a score of 2 but also result in less conservative causal effect estimates. Our linear mapping approach is thus a more accurate representation of wellbeing and results in more conservative WELLBY effects.

We conducted a sensitivity analysis using SDQ internalising scale, a combination of the SDQ Emotional Symptoms and Peer Relationships scales as an alternative measure of wellbeing. Again, we use a simple linear transformation to convert the SDQ Internalising score, between 0 and 20 to Life Satisfaction, using the formula

${LS}_{i}=2+ \frac{{20- SDQin}_{i}}{20}\times8$

where ${LS}_{i}$ is life satisfaction and ${SDQin}_{i}$ is parent reported SDQ internalising score.

The results are reported in appendix table A6. When using this wellbeing measure, the wellbeing effects of most of the risk factors are slightly larger. It is about 26% higher for having a teenage mother, 13.5% for pre-term birth, 165% for low birth weight, -20% for low height, 23% for disability and 4% for cognitive delay.

While we do have self-reported wellbeing measures for ages 11, 14 and 17- subjective happiness at ages 11 and 14 and Short Warwick Edinburgh Mental Wellbeing Score (SWEMWBS) at age 17 - the problem is that we do not yet have well-justified mapping functions from these to life satisfaction on a scale of 0-10. For example, a simple range-based linear mapping generates distributions that look different to actual life satisfaction distributions. More detail about our life satisfaction measure and mapping can be found in the paper describing the LifeSim Childhood model and its appendix [2].

**Wellbeing valuation**

The value of a WELLBY, defined as a single-unit increase in life satisfaction on a 0–10 scale for one year, is valued at £13,000 based on the HM Treasury Greenbook value. The range for this valuation is £10,000 to £16,000.

To account for inflation, the £13,000 value from 2019 has been uprated to £15,450 in 2023 levels, utilizing GDP deflators of 83.35 for 2019 and 99.06 for 2023. Our estimates can be easily adjusted to alternative valuations by dividing by 13,000 and multiplying by the chosen value. For example, the value of the wellbeing cost of preterm birth for the UK population is £1515 million (from table 2 in the paper), the upper bound value of this would be $£1515 \times\frac{16000}{13000}=£1865$ million and the lower bound of that value will be $£1515 \times\frac{10000}{13000}=£1165$ million.

**Appendix Note A5: Simulation baseline validation**

The outputs of LifeSim Childhood include a large variety of outcomes up to age 17. Due to the nature of the simulation, based on regressions, the means of the simulated outcomes will be very similar to those of the MCS data. The variation comes from the stochastic elements of the regression, coefficients from the random draw and random error terms, and random draws of the sample. Some of these output measures are available in external data, a comparison of select output measures are presented in Appendix Table A7.

We compare simulated outcome means for selected important outcomes at age 17, poor GCSEs, regular smoking, obesity, poor self-reported health, life satisfaction, cognitive ability, inpatient hospitalisation and disability. The first column presents the means in the underlying MCS data, the second column presents the means in external data where available and the final two columns provide the mean and standard deviation for our simulated data. As mentioned above the means for the MCS and simulated data are quite similar, with minor differences. Our simulated outcome means are also similar to the external data for obesity, life satisfaction and disability between 2021 and 2023. However, the simulated mean for smoking is quite different from the simulated mean for regular smoking as our model does not yet take into account the change in smoking rates between generations as cigarette use has declined.

**Supplementary Tables**

**Table A1: Descriptive Statistics**

|  | Variable | Proportion/ Mean | Standard Deviation |
| --- | --- | --- | --- |
| Country | England | 0.81 |  |
|  | Wales | 0.05 |  |
|  | Scotland | 0.09 |  |
|  | Northern Ireland | 0.04 |  |
| Region | North East | 0.04 |  |
|  | North West | 0.10 |  |
|  | Yorkshire and the Humber | 0.09 |  |
|  | East Midlands | 0.07 |  |
|  | West Midlands | 0.08 |  |
|  | East of England | 0.09 |  |
|  | London | 0.12 |  |
|  | South East | 0.15 |  |
|  | South West | 0.08 |  |
| Ethnicity | White | 0.88 |  |
|  | Mixed | 0.03 |  |
|  | Indian | 0.02 |  |
|  | Pakistani or Bangladeshi | 0.04 |  |
|  | Black | 0.03 |  |
|  | Other | 0.01 |  |
| IMD Quintile | Second | 0.19 |  |
|  | Third | 0.20 |  |
|  | Fourth | 0.19 |  |
|  | Fifth | 0.19 |  |
|  | Maternal Malaise | 0.15 |  |
|  | Smoking during pregnancy | 0.22 |  |
|  | Annual equivalised income | 18176.22 | 74.83 |
| Age 17 Adverse | Poor GCSEs | 0.41 |  |
| Outcomes | Psychological distress | 0.23 |  |
|  | Poor health | 0.12 |  |
|  | Obesity | 0.25 |  |
|  | Regular smoking | 0.14 |  |
|  | Life satisfaction (age 17) | 7.43 | 1.74 |

**Table A2: Unit Cost Summary Tables**

| **Simulated Outcomes** | **Ages Available in MCS** | **Outcome costed** | **Annual Cost per child (2023 £)** |
| --- | --- | --- | --- |
|  |  |  |  |
| Conduct disorder** | 3, 5, 7, 11, 14, 17 | Conduct disorder | 3,092 (5 to 10),  1,963 (11 to 16) |
| Persistent truancy | 11, 14 | Persistent Truancy | 943 |
| Permanent exclusion | 11, 14 | Alternative Provision | 21,848 |
| Special educational needs | 7, 11, 14 | Average cost in UK | 25,500 |
| Disability | 3, 5, 7, 11, 14, 17 | Average NHS cost for children 0 to 17 | 1,009 |
| Inpatient hospital admission | 3, 5, 7, 11, 14, 17 | Single Inpatient Episode for children | 1,587 |

*Note:*  Costs have been updated to 2023 values using annual GDP deflators from the Office of National Statistics (ONS). The GDP deflator for 2005 is 63.40 (for truancy), 2008 is 68.94 (for conduct disorder), 2016 is 78.61 (for disability), 2018 is 81.61 (for exclusion), 2020 is 87.68 (for inpatient hospitalisation), and 2023 is 99.06 (for special education needs and to uprate all other values). More details about the costs can be found in the paper describing the LifeSim Childhood model [2].

**Table A3: Results for specific outcomes (individual-level and UK population total)**

| Percentage change in probability for individuals | | | | | | |
| --- | --- | --- | --- | --- | --- | --- |
|  |  |  |  |  |  |  |
|  | Having a Teen Mother | Preterm Birth | Low Birth Weight | Low Height | Disability | Cognitive Delay |
| Poor GCSEs | 15.90 | 3.56 | 3.77 | 6.47 | 10.51 | 21.70 |
|  | (10.7- 21.1) | (0.1-7.0) | (0.7-6.8) | (3.7-9.2) | (6.0-15.1) | (18.8-24.6) |
| Psych. Distress | 0.84 | 1.29 | 1.02 | -0.28 | 2.65 | -1.07 |
|  | (-1.8-3.5) | (-0.7-3.3) | (-0.7-2.7) | (-1.9-1.4) | (0.4-4.9) | (-2.5-0.3) |
| Poor Health | 2.49 | 1.16 | 0.66 | 1.62 | 8.49 | 3.13 |
|  | (-0.8-5.8) | (-3.0-5.4) | (-1.7-3.0) | (-1.7-4.9) | (1.4-15.6) | (-0.6-6.9) |
| Obesity | 1.45 | 2.82 | -3.15 | -7.68 | 5.79 | 0.48 |
|  | (-2.7-5.6) | (-0.6-6.2) | (-5.9--0.4) | (-11.7--3.7) | (1.4-10.2) | (-1.5-2.4) |
| Regular Smoker | 7.77 | -2.40 | 2.19 | -0.46 | -5.05 | 1.59 |
|  | (2.8-12.8) | (-11.6-6.8) | (-0.4-4.8) | (-3.4-2.5) | (-10.1-0.0) | (-1.0-4.2) |
|  |  |  |  |  |  |  |
| Number of children in UK 2000 birth cohort | | | | | | |
|  |  |  |  |  |  |  |
|  | Having a Teen Mother | Preterm Birth | Low Birth Weight | Low Height | Disability | Cognitive Delay |
| Poor GCSEs | 5144 | 3942 | 3014 | 3942 | 4580 | 41204 |
|  | (3454-6834) | (57-3118) | (592-5435) | (2272-5612) | (2593-6567) | (35700-46708) |
| Psych. Distress | 272 | -168 | 815 | -168 | 1156 | -2034 |
|  | (-599-1142) | (-301-1452) | (-530-2160) | (-1183-847) | (184-2128) | (-4674-605) |
| Poor Health | 806 | 986 | 527 | 986 | 3699 | 5948 |
|  | (-273-1886) | (-1353-2389) | (-1337-2391) | (-1041-3014) | (616-6782) | (-1227-13123) |
| Obesity | 470 | -4680 | -2512 | -4680 | 2524 | 906 |
|  | (-873-1813) | (-250-2762) | (-4697--327) | (-7116--2244) | (597-4451) | (-2776-4587) |
| Regular Smoker | 2515 | -282 | 1747 | -282 | -2201 | 3023 |
|  | (904-4127) | (-5172-3031) | (-340-3834) | (-2093-1528) | (-4382--21) | (-1921-7966) |

*Note:*  Assuming a birth cohort of 679,029 births in the UK, this will amount to about 5,144 fewer children with poor GCSEs in teenage mothers were provided enough support to eliminate any potential disadvantages. The percentage change in probability is based on the number of children with each of the disadvantages in a birth cohort.

**Table A4: Results for wellbeing (individual-level and UK population total)**

| Individual wellbeing effects | | | | | | |
| --- | --- | --- | --- | --- | --- | --- |
|  |  |  |  |  |  |  |
|  | Having a Teen Mother | Preterm Birth | Low Birth Weight | Low Height | Disability | Cognitive Delay |
|  |  |  |  |  |  |  |
| Mean | 4.24 | 3.11 | 0.17 | 1.76 | 7.18 | 3.83 |
| 95% CI | (3.25 - 5.24) | (2.41 - 3.82) | (-0.41 - 0.74) | (1.07 - 2.46) | (6.13 - 8.24) | (3.16 - 4.51) |
|  |  |  |  |  |  |  |
|  |  |  |  |  |  |  |
| Population wellbeing effects | | | | | | |
|  |  |  |  |  |  |  |
|  | Having a Teen Mother | Preterm Birth | Low Birth Weight | Low Height | Disability | Cognitive Delay |
|  |  |  |  |  |  |  |
| Mean | 137,322 | 138,937 | 13,242 | 107,372 | 312,933 | 727,996 |
| 95% CI | (105,059 - 169,584) | (107332 - 170,541) | (-32,913 - 59,396) | (65,103 - 149,641) | (267,012 - 358,854) | (599,819 - 856,172) |
| (in 1000s) |  |  |  |  |  |  |

**Table A5: Results for public cost (individual-level and UK population total)**

| Individual effects (in 2023 £s) | | | | | | |
| --- | --- | --- | --- | --- | --- | --- |
|  |  |  |  |  |  |  |
|  | Having a Teen Mother | Preterm Birth | Low Birth Weight | Low Height | Disability | Cognitive Delay |
|  |  |  |  |  |  |  |
| Mean | 3,081.23 | 12,696.15 | 2,702.73 | 11,258.03 | 64,053.44 | 29,421.40 |
| 95% CI | (104.46 - 6,058.00) | (6,244.85 - 19,167.46) | (-271.80 - 5,677.26) | (4,031.10 - 18,484.96) | (37,423.27 - 90,683.61) | (9,858.59 - 48,984.21) |
|  |  |  |  |  |  |  |
|  |  |  |  |  |  |  |
| Population effects (in millions of 2023 £s) | | | | | | |
|  |  |  |  |  |  |  |
|  | Having a Teen Mother | Preterm Birth | Low Birth Weight | Low Height | Disability | Cognitive Delay |
|  |  |  |  |  |  |  |
| Mean | 99.70 | 566.40 | 215.82 | 685.71 | 2,790.15 | 5,587.84 |
| 95% CI | (3.38 - 196.01) | (277.70 - 855.10) | (-21.70 - 453.35) | (245.53 - 1,125.90) | (1,630.15 - 3,950.15) | (1,872.39 - 9,303.30) |

**Table A6: Sensitivity analysis using internalising score to measure wellbeing**

| Individual wellbeing effects | | | | | | |
| --- | --- | --- | --- | --- | --- | --- |
|  |  |  |  |  |  |  |
|  | Having a Teen Mother | Preterm Birth | Low Birth Weight | Low Height | Disability | Cognitive Delay |
|  |  |  |  |  |  |  |
| Mean | 5.34 | 3.53 | 0.45 | 1.40 | 8.82 | 4.00 |
| 95% CI | (4.44 - 6.24) | (2.69 - 4.36) | (-0.07 - 0.97) | (0.83 - 1.98) | (7.65 - 9.99) | (3.36 - 4.65) |
|  |  |  |  |  |  |  |
|  |  |  |  |  |  |  |
| Population wellbeing effects | | | | | | |
|  |  |  |  |  |  |  |
|  | Having a Teen Mother | Preterm Birth | Low Birth Weight | Low Height | Disability | Cognitive Delay |
|  |  |  |  |  |  |  |
| Mean | 172,785 | 157,277 | 35,871 | 85,413 | 384,222 | 760,402 |
| 95% CI | (143,762 - 201,809) | (120,217 - 194,337) | (-5,661 - 77,403) | (50,503 - 120,322) | (333,193 - 435,251) | (638,152 - 882,652) |
| (in 1000s) |  |  |  |  |  |  |

**Table A7: Comparison of actual and simulated outcomes at age 17**

|  | MCS | External | Simulated data | |
| --- | --- | --- | --- | --- |
|  | Mean | Mean | Mean | S.D. |
| Poor GCSEs age 17 | 0.38 |  | 0.4 | 0.08 |
| Obesity age 17 | 0.24 | 0.23 | 0.26 | 0.09 |
| Regular smoker age 17 | 0.10 | 0.02 | 0.15 | 0.09 |
| Poor health age 17 | 0.08 |  | 0.12 | 0.07 |
| Life satisfaction age 17 | 8.06 | 7.7 | 7.47 | 0.33 |
| Cognitive ability age 17 | 0.00 |  | 0.2 | 0.23 |
| Any hospitalisation age 17 | 0.17 |  | 0.22 | 0.09 |
| Disability age 17 | 0.12 | 0.11 | 0.14 | 0.07 |

*Note:*  Simulated data means are the simulated means for the randomly picked sample from the MCS. MCS means are based on means for the full imputed MCS sample. The difference in means between the MCS and simulated data is due to random sampling of the MCS data, the External figures are from various sources. Wellbeing is self-reported life satisfaction by children between 10 and 17 in response to the question “Overall, how satisfied are you with your life nowadays?” from Department for Education (2023). State of the nation 2022: children and young people’s wellbeing, Research report. Obesity is based on obesity levels at year 6 in England from Department for Education (2023). State of the nation 2022: children and young people’s wellbeing, Research report. Disability is from the Disability Census of England and Wales 2021 for children between the ages of 15 and 19 in England. Regular smoking is levels of regular smoking among 15 year olds in England in 2023 from NHS Digital Smoking, Drinking and Drug Use among Young People in England, 2023. The difference between the actual and simulated mean are due to the LifeSim Childhood model not being able to account for cohort changes in behaviour yet.

**Supplementary Figures**

**Figure A1: Simulation structure**

**Model Selection**

DAG Rules

Scientific theory

Causal evidence

Expert Consultation

Maternal age at birth

*Intervention effect*

**Data**

Millenium Cohort Study

Administrative data

Scientific literature

**Regressions**

*Coefficients from distribution*

*Random sample*

*Intervention effect*

**Simulation**

Risk factor	→	Outcome

*(ages 0 -5)	(Ages 3-17)*

**Output**

Wellbeing

Public costs

Adverse outcomes

**Figure A2: Example simple illustrative DAG for pre-term birth**

**Risk Factor**

Preterm birth

**Confounders**

Country

Region

Ethnicity

IMD quintile

Sex of child

Maternal smoking during pregnancy

Log household income

Maternal age at birth

**Outcome**
